# Not As Good as You Think? Trait Positive Emotion Is Associated with Increased Self-Reported Empathy but Decreased Empathic Performance

**DOI:** 10.1371/journal.pone.0110470

**Published:** 2014-10-29

**Authors:** Hillary C. Devlin, Jamil Zaki, Desmond C. Ong, June Gruber

**Affiliations:** 1 Department of Psychology, Yale University, New Haven, Connecticut, United States of America; 2 Department of Psychology, Stanford University, Stanford, California, United States of America; 3 Department of Psychology and Neuroscience, University of Colorado at Boulder, Boulder, Colorado, United States of America; UCLA, United States of America

## Abstract

How is positive emotion associated with our ability to empathize with others? Extant research provides support for two competing predictions about this question. An *empathy amplification* hypothesis suggests positive emotion would be associated with greater empathy, as it often enhances other prosocial processes. A contrasting *empathy attenuation* hypothesis suggests positive emotion would be associated with lower empathy, because positive emotion promotes self-focused or antisocial behaviors. The present investigation tested these competing perspectives by examining associations between dispositional positive emotion and both subjective (i.e., self-report) and objective (i.e., task performance) measures of empathy. Findings revealed that although trait positive emotion was associated with increased subjective beliefs about empathic tendencies, it was associated with both increases and decreases in task-based empathic performance depending on the target’s emotional state. More specifically, trait positive emotion was linked to lower overall empathic accuracy toward a high-intensity negative target, but also a higher sensitivity to emotion upshifts (i.e., shifts in emotion from negative to positive) toward positive targets. This suggests that trait positive affect may be associated with decreased objective empathy in the context of mood incongruent (i.e., negative) emotional stimuli, but may increase some aspects of empathic performance in the context of mood congruent (i.e., positive) stimuli. Taken together, these findings suggest that trait positive emotion engenders a compelling subjective-objective gap regarding its association with empathy, in being related to a heightened perception of empathic tendencies, despite being linked to mixed abilities in regards to empathic performance. (Word count: 242).

## Introduction

Positive emotion is a critical component for adaptive social functioning, providing meaning and enjoyment as we connect and forge bonds with others [1] [2] [3]. These salutary social effects of positive emotion suggest that feeling positive might likewise be associated with an individual’s ability to *empathically* engage with and understand others’ emotions, which is an important skill in the development and maintenance of relationships [4]. Although positive emotion and empathy are each vital to our social lives, there is a dearth of research investigating the link between them and how they might potentially intersect.

### Positive Emotion and Empathy: Two Competing Predictions

Positive emotion has been associated with many social benefits, but there is still not an entirely clear understanding on associations between positive emotionality and empathy. Empathy –attending to, sharing in, and understanding others’ subjective experiences [5] [6] [7] – critically supports an individual’s ability to socially engage with others [8]. However, in considering the extant research on the link between positive emotion and empathy, two strikingly opposed predictions emerge – namely, that positive emotion will be associated with greater levels of empathy (i.e., an *empathy amplification* hypothesis) or lower levels of empathy (i.e., an *empathy attenuation* hypothesis).

The empathy amplification hypothesis holds that positive emotionality should be associated with *greater* levels of empathy. This prediction gains support from robust associations between positive emotion and interpersonal benefits. In a broad sense, positive emotion builds social resources and fosters relationships [2]. More specific evidence consistent with this perspective includes the associations between positive emotion and enhanced relationship commitment, trust, higher-quality social interactions, and increased helping behaviors toward others [9] [10] [11]. Dispositional positive emotion is also associated with greater levels of self-reported empathic concern (i.e., feelings of care toward others in distress) and perspective-taking [4] [12] [13] [14], further suggesting that trait positive emotionality could be associated with greater empathy.

A competing perspective, which we will call the empathy attenuation hypothesis, posits that positive emotion should be associated with *lower* levels of empathy. This is based upon a line of work suggesting that positive emotion is not always socially beneficial [15]. For example, positive emotion is associated with unfavorable interpersonal outcomes that could impede normative empathic processes, including increased selfishness, stealing, stereotyping, and judgment errors in explaining the behavior of others [16] [17] [18] [19]. Positive feelings have also been associated with lower congruence of emotions between therapists and clients [20]. Furthermore, induced happiness (when compared to sadness) is associated with decreased use of theory-of-mind information in making inferences about others in a false-belief task and an interpersonal communication game [21]. Taken together, this body of work provides some evidence that positive emotion could be associated with lower levels of empathy.

Viewing these two lines of evidence on the social costs and benefits of positive emotion side-by-side produces an intriguing puzzle that the present investigation aims to address: How can positive emotion be associated with both an increased and decreased tendency to engage in prosocial processes? In recent decades, western society has become increasingly committed to better understanding how individuals can increase postitive emotion, given the wide range of benefits associated with happiness [22]. However, does positive emotion truly always confer benefits, or might it also be associated with some deleterious effects on our ability to empathize with others?

In addition, it seems possible that positive emotionality may differentially impact empathy, depending on if a target is experiencing an emotional state that is congruent (i.e., positively-valenced) or incongruent (i.e., negatively-valenced) with the perceiver’s emotions. According to the existing work on mood congruence, being in a positive emotional state makes emotion-congruent (i.e., positively-valenced) information more accessible [23], and this can impact abilities to perceive emotion in others [24] [25]. Indeed, similar emotion-congruent biases emerge in clinical populations characterized by heightened positive emotions (i.e., mania/hypomania), as this population demonstrates an increased likelihood of perceiving positive emotion in others and a decreased ability to perceive negative emotion in others [26] [27] [28]. Taken together, this body of work suggests that trait positive emotion may be associated with a heightened ability to empathize with targets expressing positive emotion; however, in regard to targets expressing negative emotion, trait positive emotion may be unrelated to, or even associated with difficulties in empathy.

### The Present Investigation: Unpacking Positive Emotion & Empathy

Empathy has often been studied in terms of its component parts (e.g., affective versus cognitive empathy). Affective empathy (or “experience sharing”) is defined as vicariously sharing in the internal states that another individual is experiencing, whereas perspective-taking (or “mentalizing”) is the cognitive form of empathy defined as inferring and understanding others’ experiences [29]. In prior research, these two forms of empathy have often been assessed using self-report measures that ask participants to rate their own empathic tendencies [13] [30] [31]. However, another related component of empathy is empathic accuracy (EA), or the ability to *accurately* detect and understand others’ emotional experiences [32] [33] [34]. Although empathic accuracy is measured in a number of ways, one standard criterion is to compare a participant’s ratings of a social target with that target’s self-report, thus assessing accuracy as agreement between two people about what one of them is experiencing [33]. Importantly, EA provides a convergent assessment of a participant’s empathic abilities that is, in important ways, more objective than standard self-report measures [32]. The use of an EA task also allows us to investigate the role of emotion-congruence, by examining participants’ empathic accuracy levels toward targets in either positive (i.e., emotion-congruent) or negative (i.e., emotion-incongruent) states.

The present investigation aims to comprehensively assess the relationship between positive emotion and two forms of empathy assessment: (1) *subjective* perceptions of empathy (i.e., self-reported empathic tendencies) and (2) *objective* measurements of empathy (i.e., performance measures of empathic accuracy abilities). This is a critical distinction for several reasons. First, subjective beliefs and objective ability do not always line up, including in the domain of empathy [35] [36] [37]. For instance, trait levels of narcissism predict individuals’ subjective beliefs that they are empathically skilled, but do not predict actual performance on objective measures of empathic ability [38]. In addition, empathy is a socially-desirable trait and therefore may be subject to self-report biases on measures that rely solely on perceptions of empathic tendencies [39]; further, it has been suggested that positive affect can prime positively-biased self-reports [40].

Therefore, the present investigation sought to systematically investigate the associations between positive emotion and empathy across both subjective and objective levels of measurement, in order to address the divergent perspectives that exist in the literature. Specifically, we employed well-validated measures of subjective (self-report) and objective (performance) empathy among a sample of adults who varied in their levels of dispositional positive emotion. We reasoned that both trait and state positive emotion might bear a potentially important association with empathy; therefore, the present study concurrently measured both trait positive emotion (measured through a standardized self-report measure) and state positive emotion (elicited via a standardized autobiographical recall task). This design enabled us to investigate the empathy amplification and empathy attenuation hypotheses in an attempt to unveil novel insights about the poorly understood relationship between empathy and positive emotion.

## Methods

### Participants

One hundred twenty one young adults (57.0% female; 47.1% Caucasian) from the Yale University or New Haven community received either course credit or were paid $10 for their participation. The mean age of the sample was 20.07 (*SD* = 3.46; range = 18–47) with an average of 13.66 years of education (*SD* = 1.39). The study was approved by the Yale University Human Subjects Committee and all participants completed a written informed consent.

### Positive Emotion Measures

#### Trait positive emotion

For our main predictor variable, we chose to focus on trait positive emotion in order to examine the relationship between temporally stable positive moods and empathy, independent of minor fluctuations in more transient and brief emotion states [41]. Dispositional positive emotion was assessed using the trait version of the Modified Differential Emotions Scale (mDES) [42], which is a well-validated measure that has been used in prior positive emotion research (e.g., [43]). The scale consists of 10 positive emotion items (i.e., amusement, awe, compassion, contentment, gratitude, hope, interest, joy, love, pride). Participants rated the degree to which they experience each emotion “in general or on average” using a 1 (*not at all*) to 5 (*extremely*) scale. A mean trait positive emotion score was created, which had high internal consistency (α = .87). For all significant results later reported, similar patterns of findings (either significant or in the predicted direction) emerged for both the mean trait positive emotion score and each individual positive emotion item on the mDES in its association with the empathy variables of interest.

#### State positive emotion

Participants completed a brief emotion induction prior to the objective empathy task to determine whether state positive emotion was associated with subsequent measures of empathy. We adapted a previously-validated autobiographical memory recall task [44] [45] [46] to elicit either a positive or neutral emotion state, which was randomly assigned across participants. For the state positive emotion elicitation, participants were asked to recall a time they felt very positive. For the state neutral emotion elicitation, participants were asked to recall a time they felt neutral. Participants were then asked to, “Go back to the time and place of the event and see the scene in your mind’s eye” and engaged in a vivid recall of the event for 60 s. Afterward, they provided a brief written narrative describing the event using the computer keypad. Participants then completed a short-form state version of the mDES [42], which asked them to rate their current experience of six positive emotions (i.e., awe, gratitude, love, pride, sympathy, interest).

### Empathic Accuracy (EA) Task

The empathic accuracy task and stimuli used in the present research were taken from prior work [33]. In this prior work, participants (whom we will refer to as “targets”) were videotaped while discussing positive and negative emotional events from their lives. After recording the videos, the targets watched their videos back and provided continuous ratings of how they had felt while discussing the event, using a 9-point sliding scale from *extremely negative* to *extremely positive*. This method allowed targets to continuously update their emotion rating throughout the film [47]. In the current study, participants watched four of these videos, which were selected to provide a range of events that varied by valence and intensity. This included two positive video clips (high and low intensity) and two negative video clips (high and low intensity). The high-intensity positive video depicted a female discussing receiving a childhood ballet scholarship (duration = 102 *s*), the low-intensity positive video described a late-night drive through the desert (duration = 117 *s*), the high-intensity negative video described the death of a parent (duration = 181 *s*), and the low-intensity negative video described a dispute with a landlord (duration = 113 *s*). Videos were blocked together by valence (i.e., two positive videos [high and low intensity] versus two negative videos [high and low intensity]), and block order was counterbalanced across participants.

### Objective Empathy Measures

#### Empathic Accuracy (EA)

Objective empathy was assessed using a previously-validated approach to measure empathic accuracy [33] [34]. In this assessment, participants watched each video and provided second-by-second online ratings of how they perceived the target to be feeling *at each moment* of the video, just as the targets themselves had previously done in rating their own emotions during the video. The text “How did this person feel while talking?” was displayed with a 9-point continuous rating scale (from *extremely negative* to *extremely positive*) on the screen directly beneath the video to allow for continuous rating while viewing the clip. Participants were instructed to adjust their rating any time they sensed a change in the target’s emotional state.

For each video, participants’ continuous ratings of how they perceived the target to be feeling were compared to the target’s own emotion ratings. As has been frequently done in prior EA research, this information was used to compute an overall EA score, which captured how accurate the participant was in continuously perceiving the target’s emotional experience throughout the video. Following the analytic strategy used in prior EA research [33], continuous emotion-rating data from both the target and perceiver were averaged across 2-*s* periods, and each 2-*s* mean served as a time-point in the subsequent analyses. Targets’ online self-ratings were correlated with perceivers’ online ratings of the target, yielding a separate coefficient referred to as an online EA score for each perceiver-clip combination [33]. All coefficients were *r*-to-*Z* transformed using Fisher’s technique, so as to be normally distributed for the analyses [48].

#### Accuracy for Affective Change (AAC)

A second objective measure of empathic accuracy involves quantifying participants’ sensitivity to positive and negative changes in the target’s self-reported emotions. Emotional changes were calculated by taking the differences in ratings in consecutive 2-*s* intervals (i.e., the first derivative in continuous ratings), and were calculated separately for the target in each video, and for each perceiver. As a concrete example, the change score at time interval *t* will be the rating at time interval *t* minus the rating at time interval (*t*-1), and will be positive if there is an increase in the rating (i.e., a positive change in the online rating), and negative if there is a decrease in the rating. If we then considered only the positive changes, and took the correlation of the participants’ positive-only changes with the target’s positive-only changes, we can calculate the participant’s differential sensitivity to positive changes. We can repeat this for negative changes, which allows for a more nuanced analysis of valence-specific sensitivity. This allows analysis of participants’ differential sensitivity to both positive changes and negative changes in each target’s emotions.

### Subjective Empathy Measures

#### Self-Reported State Empathy

Subjective state empathy was obtained by asking participants to rate the degree to which they had engaged in conscious perspective-taking toward each target during the EA task [49]. This was assessed after each video clip through two items asking how much the participant “imagined themselves in [the target’s] situation” and felt as if “they were in [the target’s] shoes” on a scale from 1 (not at all) to 7 (a great deal). These items were averaged together to create a composite perspective-taking score for each video (all αs>.86). Scores for each of the four videos were then averaged to yield an overall score of subjective state perspective-taking during the EA task, which had adequate internal validity (α = .73).

#### Self-Reported Trait Empathy

Subjective trait empathy was assessed using the well-validated Interpersonal Reactivity Index (IRI) [30]. This 28-item measure is comprised of four subscales to represent individual components of empathy, including empathic concern, perspective-taking, fantasy, and personal distress. All items are reported on a 5-point scale ranging from “Does not describe me well” to “Describes me very well.” For the purposes of the present study, we focused on the empathic concern (EC) and perspective-taking (PT) subscales, as these are two primary facets of empathy focused on in prior research. The perspective-taking scale includes seven items that capture one’s tendency to adopt the view of others in everyday life (e.g., “I sometimes try to understand my friends better by imagining how things look from their perspective”). The empathic concern subscale includes seven items to assess one’s feelings of warmth, concern, and compassion toward others in everyday life (e.g., “I often have tender, concerned feelings for people less fortunate than me”). Mean PT and EC composites were calculated, and internal consistency scores were adequate for both the PT (α = .72) and EC (α = .84) subscales.

### Other Measures of Emotionality

#### Trait Negative Affect

To confirm that our findings were specific to dispositional positive emotion (rather than trait emotionality overall), dispositional negative affect (NA) was assessed using the trait NA subscale of the mDES [42]. This measure consists of eight negative emotion items (i.e., anger, contempt, disgust, embarrassment, fear, guilt, sadness, shame). Participants rated the degree to which they experience each emotion “in general or on average” using a 1 (*not at all*) to 5 (*extremely*) scale. All items were averaged to compute a trait NA score, which had high internal consistency (α = .83).

## Results

### Data Analysis Plan

Linear regression analyses were conducted separately for each of the outcome variables: 1 measure of self-reported state empathy (i.e., perspective-taking across the EA task), 2 measures of self-reported trait empathy (i.e., empathic concern and perspective-taking subscales of the IRI), and 2 measures of objective empathy (i.e., empathic accuracy and accuracy for affective change). The measures of objective empathy were calculated separately for each of the four videos viewed by participants: (1) high-intensity negative event, (2) high-intensity positive event, (3) low-intensity negative event, and (4) low-intensity positive event. All reported *p* values are two-tailed and reported beta values are standardized coefficients.

### State Positive Emotion and Empathy

A t-test revealed that the state positive emotion induction task was effective in eliciting the intended emotion state, as the positive condition reported significantly greater state positive emotion after the induction task as compared to the neutral condition, (*t*(117) = 8.98, *p*<.001). The relationship between state positive emotion (vs. neutral emotion comparison condition) and empathy was then examined using a series of one-way ANOVAs to test if any of the subjective or objective empathy variables differed across the emotion conditions of the recall task. No differences between the positive and neutral conditions emerged on any measures of empathy. Thus we have opted not to report these state positive emotion results in further detail in the present study, and collapsed across the positive and neutral conditions for the remainder of our analyses.

### Trait Positive Emotion and *Subjective* Empathy

Trait positive emotion was associated with heightened perceptions of one’s own empathic tendencies on the subjective empathy measures. More specifically, trait positive emotion was significantly associated with greater self-reported trait empathic concern (β = 0.33, *t*(119) = 3.81, *p*<.001) and perspective-taking (β = 0.38, *t*(119) = 4.53, *p*<.001) on the subscales of the IRI. Further, positive emotion was positively associated with self-reported perspective-taking at the state-level as well: that is, the extent to which participants reported engaging in perspective-taking across the four videos of the EA task (β = 0.37, *t*(119) = 4.36, *p*<.001). Parallel results emerged for each video separately, except the low-intensity negative video, on which state perspective-taking shared no relationship with trait PA; therefore, toward this one target, trait PA did not predict self-perceptions of having taken the targets’ perspective.

### Trait Positive Emotion and *Objective* Empathy

#### Empathic Accuracy (EA)

When examining our objective measure of empathic accuracy performance (i.e., the ability to track moment-to-moment fluctuations in a target’s emotion experience over time), trait positive emotion was associated with lower empathic performance toward the high-intensity negative target, and unrelated to empathic performance toward the other three targets. More specifically, trait positive emotion was associated with lower EA toward the targets from the two negative films (β = −0.26, *t*(119) = −2.90, *p*<.01), but when running EA toward each of these targets separately, trait positive emotion was only significantly associated with lower objective empathy toward the target describing a high-intensity negative event (β = −0.23, *t*(119) = −2.63, *p*<.01). By contrast, trait positive emotion did not track with EA for positive events (β = −0.07, *t*(119) = −0.73, *p*>.05). *Z* transformations were used to compare the strength of correlations of trait positive emotion and EA on the high-intensity negative video versus the other three videos collapsed [50]. This analysis revealed that trait positive emotion was associated with significantly lower EA toward the high-intensity negative target, as compared to the other three targets (*Z* = −3.95, *p*<.01).

#### Accuracy for Affective Change (AAC)

When examining the objective empathy measure of accuracy for affective change (i.e., sensitivity to shifts in a target’s emotion), trait positive emotion was marginally associated with increased sensitivity to positive changes in the targets’ emotions across all four videos (β = 0.0012, *t*(119) = 1.80, *p* = .07); specifically, this is driven by strong associations between trait positive emotion and sensitivity to positive changes in the targets describing the high-intensity positive event (β = 0.0035, *t*(119) = 2.73, *p*<.01) and low-intensity positive event (β = 0.0019, *t*(119) = 2.06, *p*<.05). Thus, trait positive emotion tracks increased sensitivity to upshifts in positive emotion regarding positive events, but not overall empathic accuracy for positive events (previous section), possibly because of the inclusion of negative changes. The only other significant association was between trait positive emotion and sensitivity to negative changes in the target describing the low-intensity negative event (β = 0.0023, *t*(119) = 2.05, *p*<.05).

### Secondary Analysis: Other Measures of Emotionality

Follow-up analyses were conducted to determine if the observed relationships between trait positive emotion and empathy were due to other measures of emotionality (i.e., trait negative emotion). All significant results held when re-analyzed while statistically controlling for this covariate, except for the sensitivity findings to positive changes in the low intensity positive video and negative changes to the low intensity negative video, which could be due to the fact that the sensitivity analyses are much noisier (see Table 1 for all results when controlling for trait negative emotion). These secondary analyses suggest that there is something specific about trait positive emotion that is uniquely related to empathy, whereas trait negative emotion is not. Furthermore, when trait NA was tested on its own, it was not significantly associated with any of the empathy variables, with the exception of subjective trait perspective-taking on the IRI (β = 0.19, *t*(119) = 2.16, *p*<.05), in that individuals high in negative emotionality appear to report engaging in more perspective-taking in their daily lives.

**Table 1:** Standardized Coefficients for Trait Positive Emotion as Predictor of Empathy Controlling for Other Measures of Emotionality.

Outcome Measure	β (Controlling for Trait NA)
Subjective trait empathic concern (IRI)	0.33**
Subjective trait perspective-taking (IRI)	0.38**
Subjective state perspective-taking (across all videos)	0.37**
Objective EA (across positive videos)	−0.07 n.s.
Objective EA (across negative videos)	−0.26**
Objective EA (high negative video)	−0.24**
Objective EA: Positive changes (high positive video)	0.00038*
Objective EA: Positive changes (low positive video)	0.0018 n.s.
Objective EA: Negative changes (low negative video)	0.0009 n.s.

*Note.* All standardized coefficients reported are when controlling for trait NA as a covariate.

EA = Empathic Accuracy; IRI = Interpersonal Reactivity Index. *p<.05, **p<.01.

## Discussion

The present investigation aimed to reconcile an empirical paradox regarding the relationship between trait positive emotion and empathy, using empathic assessments that tapped into both subjective beliefs (i.e., self-reported perceptions of one’s own empathic tendencies) and objective abilities (i.e., performance on an empathic accuracy task). The current findings suggest that trait positive emotion is associated with a subjective-objective gap in regards to empathy. On the one hand, trait positive emotion is linked to higher subjective beliefs about one’s own levels of empathy; in other words, it is associated with a self-reported perception of greater empathic tendencies. However, using more objective task-based measures of empathy (i.e., EA, AAC), we found that trait positive emotion was associated with a more complex portrait of relative increases and decreases in empathic ability. More specifically, objective empathic ability appears to be in part contingent on the degree to which the target’s emotional state is congruent with the participant’s trait positive emotionality. In support of this, trait positive emotion was associated with *lower* empathic performance in continuously tracking the emotions of a target experiencing high-intensity negative (i.e., incongruent) emotions, and was unrelated to overall empathic accuracy toward the other three targets. However, trait positive emotion was also linked to a *higher* sensitivity in specifically detecting upshifts in positive (i.e., congruent) emotion among positive targets. Taken together, these findings suggest the potential role of emotion-congruence in the relationship between trait positive emotion and empathic abilities.

Prior work on mood congruence suggests that positive affect often facilitates the accessibility and recall of positive information [22]. Perhaps individuals high in trait positive emotionality experience more difficulties processing and identifying a target’s emotions when it is highly incongruent with their own trait affect (as seen with the high-intensity negative target) but are particularly sensitive to shifts in emotion that are congruent with their overall trait affect (as seen with greater accuracy in detecting emotion upshifts in positive targets). Although empathic accuracy (i.e., accuracy in the continuous tracking of others’ emotions) is likely more revealing about overall empathic performance, our affective change accuracy analysis provides further insight into what specific information participants are using to rate a target’s emotions. These AAC findings seem to suggest one unique way that emotion rating may be altered by trait positive emotion is through sensitivity to upshifts in emotion.

### 

#### Implications for Positive Emotion Research

In a broad sense, the current findings help to reconcile two conflicting hypotheses regarding the social processes associated with positive emotion. First, it appears that method of assessment (i.e., reliance on subjective versus objective measures) is one factor that could help to explain why positive emotion has been linked to both adaptive and maladaptive social outcomes in prior research. More generally, these findings lend support for the overarching idea that teasing apart such distinctions can advance our broader understanding of positive emotion. Future studies examining how positive emotion influences interpersonal outcomes – while incorporating both subjective and objective markers of performance to reduce potential sources of measurement error – may continue to produce novel insights about the multifaceted nature of positive emotion.

#### Implications for Empathy Research

The current findings also highlight important considerations about the assessment of empathy, as well as assessment more generally in psychological research. The identification of a belief-ability gap augments prior research suggesting that subjective measures often do not map onto objective markers of performance, and the two can even be inversely related [35] [38]. It seems critical that this be continually taken into account in the interpretation of empathy research, as well as in other performance domains, particularly if subjective self-report measures are the only method relied upon. The current findings underscore the importance of assessing objective empathy, in addition to subjective self-reports, in order to most fully capture participants’ empathic abilities.

In addition, the current findings suggest that emotion-congruence should be taken into account when examining the effects of emotionality on empathy. As prior work has noted, a participant’s emotions may impact empathic ability in different ways depending on if the target is displaying an emotion -congruent or -incongruent state [25]. Therefore, measuring empathy toward a range of targets in both positive and negative states can uncover the contexts in which trait positive emotion may uniquely help, and hinder, empathic performance. The present findings may also help to advance our understanding of empathic deficits experienced by specific clinical populations that experience disruptions in positive emotion. For example, given the patterns of empathic performance that emerged, future investigation is warranted to determine if decreased empathic accuracy toward others’ high-intensity negative emotions may be a mediating factor that explains why individuals who exhibit positive emotion persistence (e.g., during mania in bipolar disorder) develop strained social relationships [51] [52].

### Limitations & Future Directions

There are several caveats of the present study that should be noted. First, a potential concern is that our measure of objective empathy, the EA task, uses standardized videos of strangers discussing life events and directly instructs participants to attend to the emotions of someone else. In the present study, we only featured four female targets in these videos, which could introduce confounds due to characteristics specific to those targets (e.g., appearance, etc.). This method strengthened our study in that it afforded us more control and standardization [33], but may not reflect one’s empathic accuracy when they spontaneously perceive the emotions of others in everyday life. Future research could employ dyadic paradigms with the aim of exploring the effects of positive emotion on empathy during in-vivo exchanges. Second, some primary variables of interest (e.g., trait positive emotion, subjective empathy) were assessed using only self-report measures. The present results highlight the importance of relying on multiple methodologies. For example, in assessing trait PA, future studies should aim to include psychophysiological and additional behavioral measures in order to capture a richer assessment of emotion. Third, the present results are correlational in nature, and therefore, causality cannot be inferred regarding the relationship between positive emotion and empathy. However, our findings do suggest an association between trait positive emotion and a subjective-objective gap in relation to empathy. Although the present research found that a brief experimental induction of positive emotion did not impact subsequent ratings or performance on the empathy outcomes measured, it will be important to further examine whether other induction modalities of state positive emotion might directly impact empathy in an effort to establish a more causal relationship. Perhaps our autobiographical recall induction was not powerful enough to sustain a positive (or neutral) state while participants watched the EA videos, which could have potentially been emotion-eliciting. Furthermore, it is possible that specific types of positive emotion would have an effect on empathy – for example, perhaps the induction of specific discrete positive emotions uniquely relevant to social engagement and connection with others (e.g., compassion, gratitude) may help to uncover such relationships.

In the absence of observing a direct relationship between state positive emotion and empathy, we feel it important to underscore the possibility that trait positive emotion, by contrast, may simply have a more direct association on the perception of emotion in others. Specifically, perhaps higher trait positive emotion experienced across contexts may build more long-term perceptual biases in perceiving others’ emotion, in that repeated experiences of positive emotion are what leads to more consistent and robust patterns of empathic accuracy performance as observed in the current research. Indeed, dispositional differences in emotionality can influence where one’s attention is selectively focused in situations, and in turn, likely guide social perceptions and outcomes [53]. Lastly, it will be important for future research to further explore why trait positive emotion is associated with lower EA toward high-intensity negative targets. For example, are individuals high in trait positive emotion less able to relate to this target’s emotionality and therefore exhibit less EA toward them? Do they assume perceived emotional similarity and therefore overestimate the target’s positive emotion? It will be critical to unpack such possibilities through further investigation.

### Concluding Remarks

The present research tested two competing hypotheses regarding positive emotion and empathy (i.e., the empathy amplification and attenuation hypotheses), and initially appeared to provide partial support for each of these seemingly disparate perspectives. In support of empathy amplification, trait positive emotion was associated with greater empathy according to subjective self-reports from the participant. In line with this, trait positive emotion was associated with one form of greater objective empathy in detecting emotion-congruent (i.e., positive) upshifts in affect among positive targets. In contrast to these findings, positive emotion was not significantly associated with greater empathy on all other objective measures of performance, and in fact, it was associated with poorer overall empathic accuracy in perceiving the emotions of a target in high distress, thereby lending some support for the empathy attenuation perspective. These findings highlight the possibility of a belief-ability gap: although trait positive emotion is linked to greater beliefs about one’s own empathy, it is not associated with most objective performance markers on an empathic accuracy task. However, on the markers with which it does share a significant relationship, trait positive emotion can be associated with either increased *or* decreased objective empathic performance, and emotion-congruence with the target’s state seems to play an important role in this.

This discrepancy between beliefs and abilities is consistent with a substantial literature that has contrasted subjective and objective measures in other performance domains [35]. It has been found that subjective self-perceptions often do not accurately reflect objective performance, and this incongruence has even been identified in regards to interpersonal perception and mind-reading [36] [38] [54]. The current findings suggest that trait positive emotion could be one driving factor that promotes some divergence between beliefs about empathic tendencies and actual empathic accuracy performance.

The current findings suggest that trait positive emotion is associated with both strengths and weaknesses in the ability to accurately read others. It makes intuitive sense that we see greater empathic abilities emerge in the face of positive targets, given both emotion-congruence accounts [22] and the notion that humans are primarily driven to maintain positive feelings and therefore may be more willing to empathize with such targets [55]. However, it perhaps takes more sacrifice to “drop down” and focus on another person’s high-intensity negative emotions, and this may be particularly difficult to do if this state is highly incongruent with an individual’s emotional disposition. In line with this, it was only when faced with another person in distress that high trait positive emotion was associated with less ability to be empathically accurate. Yet this is of note because individuals who are experiencing intense negative emotions may often be those who need support and empathy the most. The present findings do not speak to the possibility of if individuals high in trait positive emotion would be less willing or able to provide support and assistance. However, if a global positive emotionality perhaps leaves us less willing to shift out of our own state and be empathically accurate toward others facing negative experiences, deleterious social consequences could follow suit. In order to uncover such possibilities, future research is warranted to further parse apart how positive emotion can uniquely impact our subjective beliefs and objective abilities and the potential mechanisms that may underlie this relationship.

## Supporting Information

File S1 EEW_ANOTATED.sav is the dataset with scores of trait emotion, subjective empathy, and objective empathy. EARatings_ForEachTarget.xls contains the continuous emotion ratings from each video’s target (averaged across every 2 seconds). Targets are listed across the top, which include: high negative, low negative, high positive, and low positive. The other four EARatings excel files are the participants’ (from the current sample) continuous emotion ratings of how they perceived each target to be feeling from second-to-second. Participant IDs are listed along the top of each file.
(ZIP)
